# Stretch‐Induced Ordering of Prochiral Dimethyl Sulfoxide in Anisotropic Hydrogels Analysed by ^1^H and ^2^H Nuclear Magnetic Resonance

**DOI:** 10.1002/cphc.202400731

**Published:** 2024-11-27

**Authors:** Stuart J. Elliott, Philip W. Kuchel, Thomas R. Eykyn

**Affiliations:** ^1^ Molecular Sciences Research Hub Imperial College London London W12 0BZ United Kingdom; ^2^ School of Life and Environmental Sciences University of Sydney Sydney NSW 2006 Australia; ^3^ School of Biomedical Engineering and Imaging Sciences King's College London St Thomas' Hospital London SE1 7EH United Kingdom

**Keywords:** anisotropic hydrogels, prochiral DMSO, ^1^H/^2^H nuclear magnetic resonance, dipolar/quadrupolar splittings, S−Me bond order parameters, ^2^H imaging

## Abstract

Nuclear spins in small molecules dissolved in stretched hydrogels typically have population‐averaged residual interactions. The nuclear magnetic resonance (NMR) spectra of these systems often show additional peaks and splittings compared with free solutions. Residual dipolar couplings (RDCs) and quadrupolar couplings (RQCs) are observed for guest ^1^H and ^2^H nuclear spins, respectively. Dimethyl sulfoxide (DMSO) is an exquisitely sensitive probe of such biologically relevant environments since it is prochiral and becomes effectively chiral when embedded in anisotropic gelatin‐based hydrogels. Measured ^1^H RDCs and ^2^H RQCs were used to estimate bond order parameters over a wide range of stretching extents. At the largest extent of stretching, the ^2^H splittings were −73.0 and −9.4 Hz, similar to those found for guest molecules in liquid crystals. Inhomogeneous line broadening of the ^2^H resonances was related to the size of the RQC due to a spatial distribution of RQCs, which was revealed using a one‐dimensional slice selective imaging experiment along the stretching direction. ^1^H NMR spectra exhibited homogeneous line broadening, with resonance integrals that indicated concealed multiplet structure. Understanding molecular bond ordering in mechanically oriented environments provides a conceptual framework for investigating more complex systems including zeolites and those found *in vivo*.

## Graphical abstract

Residual couplings in the ^1^H and ^2^H nuclear magnetic resonance spectra of prochiral dimethyl sulfoxide dissolved in an anisotropic gelatin gel showed linearity with stretching extent and were used to determine specific bond ordering. *SpinDynamica* simulations matched experimental ^2^H spectra using a Gaussian distribution of S‐Me order parameters supported by a 1D slice‐selective imaging experiment along the stretching direction.

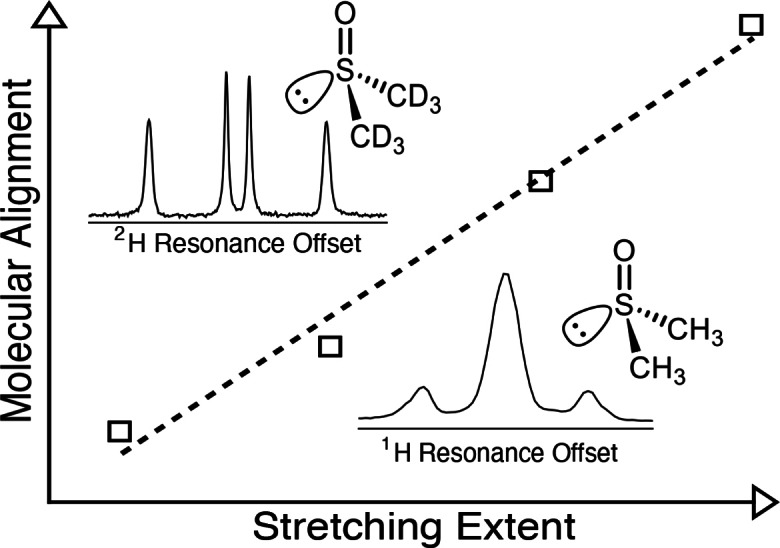

## Introduction

Stretched and compressed hydrogels provide a reproducible and controllable way of creating a macroscopic alignment medium that can mimic conditions in tissues and cells.^[1]^ Gelatin is derived from collagen, the main structural protein of the extracellular matrix, which is also abundant in connective tissue. There is a high concentration (~30 %) of prochiral glycine, however, it is intrinsically chiral due to the l‐enantiomers of the other constituent amino acids as well as the right‐handed turn of the peptide chains that form a triple helical bundle, which is also right‐handed. The chiral medium has a net alignment once stretched in a collective manner. The secondary structure of the triple helix also has its own (pro)chiral‐alignment effect. When stretched or compressed, these hydrogels yield anisotropic chiral environments, which provide a means of discriminating between the pro‐R/S enantiomers of prochiral molecules by ^1^H and ^2^H nuclear magnetic resonance (NMR).^[2]^ Chiral liquid crystals have also been used for prochiral resolution of small molecules,^[3]^ including ^2^H at natural abundance,^[4]^ however, the extent of alignment is not as readily tuneable as with gels.

The two methyl groups in dimethyl sulfoxide (DMSO) are prochiral due to the sulphur lone pair of electrons (*cf*. acetone which is achiral). The ^2^H NMR spectrum of DMSO in isotropic solution presents as a singlet, which in a stretched hydrogel resolves into a pair of quadrupolar doublets that can be assigned to one or other of the methyl groups due to the anisotropic chiral environment.^[4]^ The quadrupolar splitting is a consequence of molecular bond ordering in the mechanically distorted hydrogel;^[5]^ which occurs because the intrinsic electric quadrupole moment of the guest nuclear spin *I*>1/2 interacts with molecular‐length‐scale electric field gradients (EFGs) in the anisotropic host medium.^[6]^

Molecular bond ordering also manifests as residual dipolar couplings (RDCs) in ^1^H NMR spectra of small molecules dissolved in anisotropic media.^[7]^ A 1 : 2 : 1 triplet is observed in the ^1^H NMR spectrum of monodeuterated methane (CH_3_D) dissolved in a liquid crystal.^[8]^ Full spectral assignment of the natural product artemisinin has been achieved via one‐bond ^13^C−^13^C RDCs at natural abundance in a polymethylmethacrylate (PMMA) gel.^[9]^

Methyl groups typically show smaller values of ^2^H RQCs *vs*. non‐mobile groups, which may be due to an averaging effect that results from rapid rotation of the moiety. Nonetheless, methyl groups are exceptional probes of molecular structure in biomolecular NMR.^[10]^ RDCs and RQCs extracted from freely rotating groups can be interpreted in terms of molecular configuration.^[11]^

In this Article, we report a detailed analysis of the ^1^H and ^2^H NMR spectra of prochiral DMSO in anisotropic hydrogel samples stretched to different extents. The alignment of DMSO is defined in terms of S−Me bond order parameters. Measurement of ^2^H relaxation time constants and numerical simulations of experimental ^2^H NMR spectra revealed the effects of a statistical distribution of S−Me bond order parameters. Differential line broadening due to a small extent of sample inhomogeneity along the direction of hydrogel stretching was revealed by a slice selective ^2^H *z*‐imaging experiment. The current work extends previous investigations of prochiral molecules in aligned gels.

## Experiments

### Sample Preparation

For 40 % *w*/*v* hydrogels, 0.3 g of granulated bovine gelatin was dissolved in 0.7 mL of phosphate buffered saline (PBS) reconstituted from a tablet (*Merck*, Darmstadt, Germany), 137 mM NaCl, 3 mM KCl and 10 mM phosphate with 60 mM NaOH added (to neutralize to physiological pH=7.4 at 25 °C) in a 1 mL Eppendorf centrifuge tube. Samples were prepared with 5 % DMSO‐*h*_6_ and 5 % DMSO‐*d*_6_ or 5 % acetone‐*d*_6_ and 5 % DMSO‐*d*_6_. The gelatin solution was placed in a heated block at 80 °C for ~30 min (until thoroughly dissolved) and centrifuged at ~2000×g for 30 s to remove air bubbles. The gelatin solution was drawn into a ~25 cm long silicone rubber tube (*Altec*, St Austell, United Kingdom; 3.5 mm o.d., 2.5 mm i.d.) via an attached 1 mL plastic syringe. The end of the tube was sealed with a custom‐made Luer plug, taking care to avoid introducing air bubbles. The loaded silicone rubber tube was inserted into the bore of an 18 cm long bottomless 5 mm o.d. glass NMR tube (*Wilmad*, Vineland, NJ, USA). The samples were cooled to room temperature (~20 °C) before manually stretching.^[12]^

### Data Acquisition and Processing

All experiments were performed on a *Bruker Biospin* NMR spectrometer with 9.4 T vertical bore magnet (^1^H and ^2^H nuclear Larmor frequencies, 399.47 and 61.31 MHz, respectively) at 25 °C. The ^2^H lock was turned off, and to improve ^2^H detection sensitivity, the X‐transmitter amplifier was routed via the ^2^H preamplifier, and onto the X‐channel of the 5 mm BBO probe. ^1^H NMR spectra were acquired with a spectral width of 13.22 ppm, 4096 time domain data points, and an acquisition time of 0.39 s. ^2^H NMR spectra were acquired with a spectral width of 20 ppm, 4096 time domain data points and an acquisition time of 1.67 s. Spectra were derived from summation of four free induction decays (FIDs) with an interval of 2 s between each. Exponential line broadening with a factor of 0.3 Hz was used prior to Fourier transformation, and subsequent phase and baseline correction.

### Slice Selective ^2^H z‐Imaging

Slice selective imaging was performed along the *z*‐direction of the NMR tube using selective excitation with a 6.18 ms duration Q5 pulse simultaneously applied with a *z*‐gradient with an amplitude of 5.35 mT cm^−1^ (100 % amplitude corresponding to 34967.6 Hz cm^−1^ for ^2^H). The bandwidth of the pulse was 1 kHz yielding a slice thickness of 0.29 mm. Imaging of the entire active region of the radiofrequency (*rf*) coil was achieved by stepping the offset frequency of the pulse from −35 to +35 kHz in increments of 1 kHz. Slice selection was chosen over phase encoding as each slice had the same phase and therefore the one‐dimensional image could be processed by Fourier transformation along the acquisition dimension without phase distortion. Evolution of the quadrupolar coupling during the pulse was identical for all slices since the *rf*‐pulse length was constant.

### Spectral Simulations

The *Mathematica*‐based NMR software package *SpinDynamica* (version 3.7.0) was used for all simulations of NMR spectra.^[13]^ The simulated ^1^H NMR spectrum was obtained using the dipolar Hamiltonian:^[14]^

(1) $${\hat{H}}_{D}=\sqrt{6}\sum_{\lambda }\sum_{i<j}D_{ij}^{\lambda }\;{\hat{T}}_{ij}^{20},$$

where ${\hat{T}}_{ij}^{20}$ is a second‐rank spherical tensor operator:

(2) $${\hat{T}}_{ij}^{20}=\frac{3{\hat{I}}_{iz}{\hat{I}}_{jz}-{\hat{I}}_{i}{\hat{I}}_{j}}{\sqrt{6}},$$

and ${\hat{I}}_{i}$ and ${\hat{I}}_{iz}$ are the total and *z*‐angular momentum operators for spin *i*, respectively. $D_{ij}^{\lambda }$ is the RDC constant:

(3) $$D_{ij}^{\lambda }=S_{0}^{\lambda }\;d_{00}^{2}\left(\beta_{PF}^{D}\right)\;\omega_{D},$$

where $S_{0}^{\lambda }$ is the S−Me bond order parameter for enantiomer λ , $d_{00}^{2}\left(\beta_{PF}^{D}\right)$ is the central element of the second‐rank reduced Wigner rotation matrix, $\beta_{PF}^{D}$ is the angle between the *z*‐axes of frames *P* and *F* (see below) and $\omega_{D\;}$ is the dipolar coupling constant:^[15]^

(4) $$\omega_{D}=-\frac{\mu_{0}\hbar \gamma_{i}\gamma_{j}}{4\pi r_{ij}^{3}},$$

and $\gamma_{i}$ is the magnetogyric ratio of nuclear spin *i* and *r_ij_* is the internuclear distance between spins *i* and *j*.

The simulated ^2^H NMR spectrum was obtained using the quadrupolar Hamiltonian:^[16]^

(5) $${\hat{H}}_{Q}=\frac{1}{\sqrt{6}}\sum_{\lambda }\sum_{i}B_{i}^{\lambda }\;{\hat{T}}_{i}^{20},$$

where ${\hat{T}}_{i}^{20}$ is also a rank‐2 spherical tensor operator:

(6) $${\hat{T}}_{i}^{20}=\frac{3{\hat{I}}_{z}^{2}-I\left(I+1\right)\hat{𝕀}}{\sqrt{6}},$$

and $\hat{𝕀}$ is the identity operator. $B_{i}^{\lambda }$ is the RQC constant:

(7) $$B_{i}^{\lambda }=S_{0}^{\lambda }\;d_{00}^{2}\left(\beta_{PF}^{Q}\right)\;\omega_{Q},$$

where the quadrupolar coupling frequency $\omega_{Q}$ of the ^2^H nucleus is:^[17]^

(8) $$\omega_{Q}=\frac{3eQV_{zz}}{2I(2I-1)\hbar },$$

and Q is the electric quadrupole moment of the ^2^H nucleus and $V_{zz}$ is the electric field gradient (EFG) at the ^2^H nucleus. The EFG tensor is assumed to be axially symmetric with a biaxiality of η=0 .^[18]^ The quadrupolar coupling constant, often defined as $C_{Q}=eQV_{zz}/h$ , is a factor of 3/2 times the value given in Eq. (8) for an *I*=1 nuclear spin, namely, $\omega_{Q}/2\pi =3C_{Q}/2$ .

The central element of the rank‐2 reduced Wigner rotation matrix is:

(9) $$d_{00}^{2}\left(\beta_{PF}^{\zeta }\right)=\frac{3Cos{\left(\beta_{PF}^{\zeta }\right)}^{2}-1}{2},$$

where $\beta_{PF}^{\zeta }$ is the angle between the *z*‐axis of the frame of the principal axis system (*P*) and a frame with its principal axis coincident with the S−Me bond (*F*) for interaction ζ (*D* or *Q*).^[19]^ For the ^1^H−^1^H dipolar interaction, Eq. (9) is equal to −1/2 for the case of a CH_3_ group since the principal axis of the *P* frame is the vector connecting two protons with $\beta_{PF}^{D}=90^{\circ }$ . For the ^2^H quadrupolar interaction, Eq. (9) is approximately equal to −1/3 for a CD_3_ moiety since it is assumed that the principal axis of the *P* frame is coincident with the C−D bond with $\beta_{PF}^{Q}=70.5^{\circ }$ , the dihedral angle of a tetrahedron.

In reality, there is a distribution of order parameters for enantiomer λ that we assumed to be Gaussian:

(10) $$g^{\lambda }\left(x\right)=S_{0}^{\lambda }\;exp\left(-\frac{{\left(x-S_{0}^{\lambda }\right)}^{2}}{2{\left(\sigma^{\lambda }\right)}^{2}}\right),$$

such that $g^{\lambda }\left(S_{0}^{\lambda }\right)=S_{0}^{\lambda }$ and $\sigma^{\lambda }$ is the square root of the variance of a Gaussian distribution centred at $S_{0}^{\lambda }$ . The ^2^H NMR spectral simulations were repeated for values of *x* from $S_{0}^{\lambda }-2\sigma^{\lambda }$ → $S_{0}^{\lambda }+2\sigma^{\lambda }$ in increments of $S_{0}^{\lambda }/250$ . The resulting ^2^H NMR spectrum was the sum of individual spectra simulated over the range $g^{\lambda }\left(x\right)$ . In *SpinDynamica*, the function *EnsembleAverage* was used to achieve the desired Gaussian distributions.

### Quadrupolar Relaxation

The dominant quadrupolar relaxation mechanism is the result of fluctuating interactions between the electric quadrupole moment of the *I*=1 nuclear spin and EFGs at the ^2^H nucleus imposed by the matrix of the gel. Longitudinal ($T_{1}$ ) and transverse ($T_{2}$ ) nuclear spin relaxation rate constants are:

(11a) $$T_{1}^{-1}=\frac{1}{30}\omega_{Q}^{2}\;\left(J\left(\omega_{0}\right)+4J\left({2\omega }_{0}\right)\right),$$

(11b) $$T_{2}^{-1}=\frac{1}{60}\omega_{Q}^{2}\;\left(3J\left(0\right)+5J\left(\omega_{0}\right)+2J\left({2\omega }_{0}\right)\right),$$

where $J\left(m\omega_{0}\right)$ is the spectral density function:^[20]^

(12) $$J\left(m\omega_{0}\right)=\frac{\tau_{e}}{1+{\left(m\omega_{0}\tau_{e}\right)}^{2}},$$

where *m* is an integer (0, 1 or 2), $\omega_{0}$ is the ^2^H nuclear Larmor frequency and $\tau_{e}$ is the effective correlation time of molecular tumbling. The latter has contributions from both the rotational correlation time ($\tau_{c}$ ) for overall molecular reorientation and a rotor correlation time describing methyl group rotation. The model‐free approach could have alternatively been used to described ^2^H relaxation in DMSO‐*d*_6_ due to the presence of two distinct motional timescales.^[21]^

## Results

### NMR Spectra

**Figure 1:**
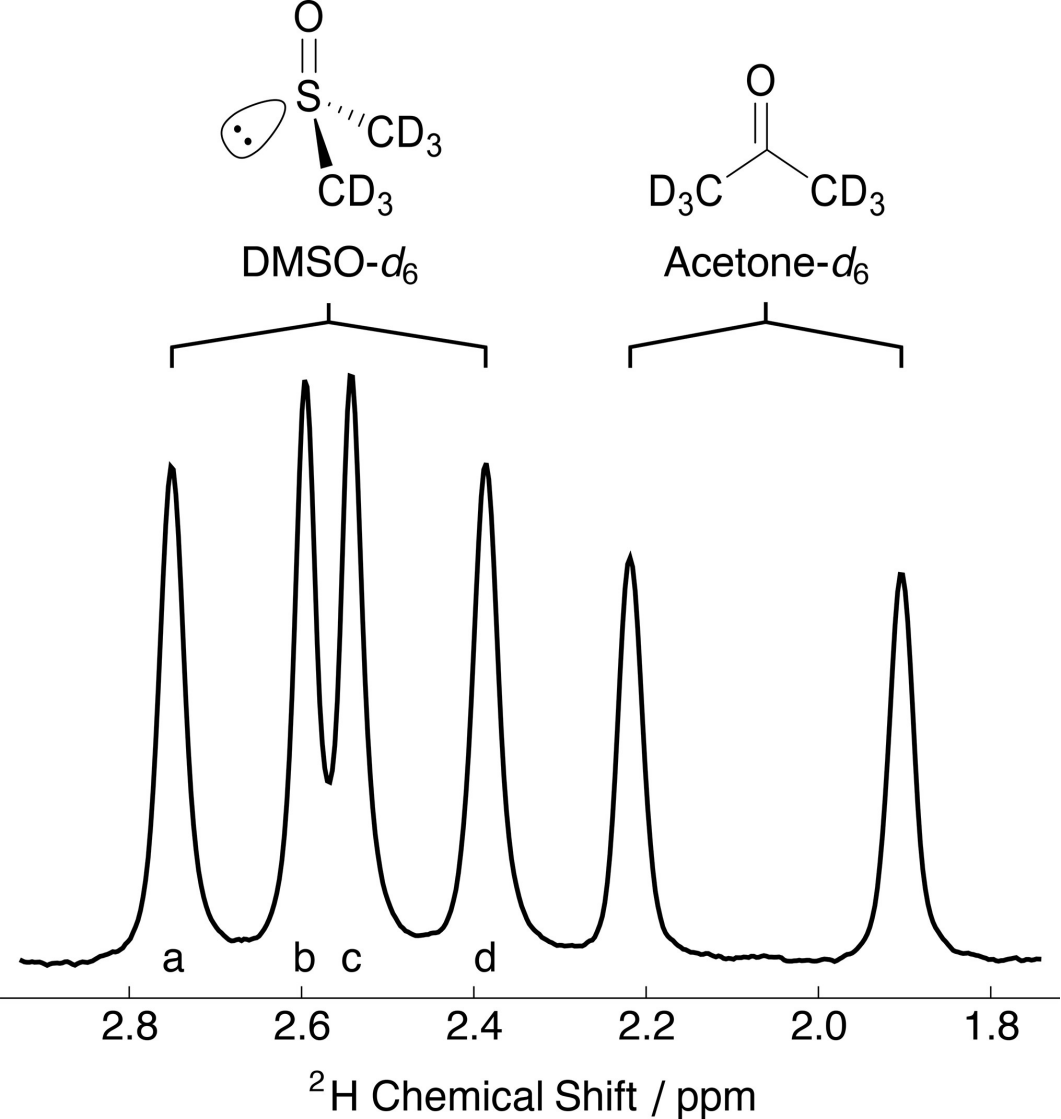
Relevant portion of the experimental ^2^H NMR spectrum of a sample of 5 % DMSO‐*d*_6_ and 5 % acetone‐*d*_6_ dissolved in a stretched gelatin gel (extent=6 cm; 1.3‐fold) acquired at 298 K.

Figure 1 shows the relevant portion of the ^2^H NMR spectrum obtained from a sample of 5 % DMSO‐*d*_6_ and 5 % acetone‐*d*_6_ dissolved in a gelatin gel that was stretched by 6 cm over the 18 cm NMR tube (1.3‐fold). DMSO‐*d*_6_ yielded two doublets, while acetone‐*d*_6_ revealed only a single doublet. The ^2^H splitting observed for acetone‐*d*_6_ (−19.3 Hz) was similar to the outer ^2^H splitting of DMSO‐*d*_6_ (−22.4 Hz).

**Figure 2:**
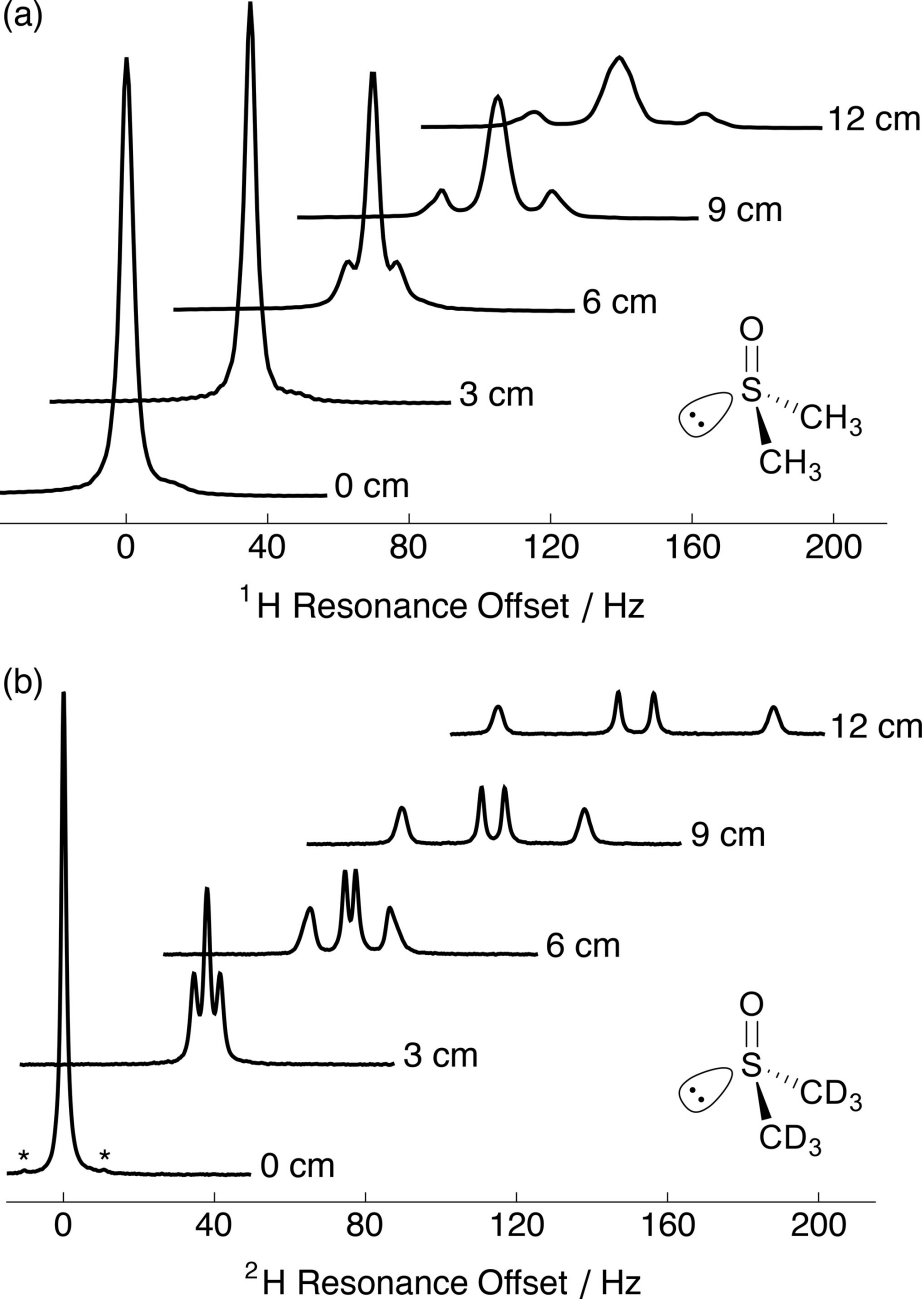
Relevant portions of the (a) ^1^H and (b) ^2^H NMR spectra of 5 % DMSO‐*h*_6_ and 5 % DMSO‐*d*_6_ dissolved in a gelatin gel acquired at 298 K under several extents of stretching. *^13^C satellite peaks (|^1^ *J*_CD_|=21.0 Hz).

Figure 2 shows the relevant portions of the experimental NMR spectra: (a) ^1^H and; (b) ^2^H of 5 % DMSO‐*h*_6_ and 5 % DMSO‐*d*_6_ dissolved in the hydrogel and stretched to different extents. Spectra from the relaxed gel (extent=0 cm) showed no resolved (residual) coupling. Introducing a small anisotropy by gel stretching (extent=3 cm) did not produce an observable ^1^H−^1^H peak splitting, while the outer ^2^H splitting was partially resolved (triplet appearance). With further stretching, the ^1^H multiplet structure emerged and the outer peaks were well‐resolved when stretched beyond 9 cm (1.5‐fold). Both ^2^H doublets became resolved at a smaller stretching extent (6 cm, 1.3‐fold). The residual dipolar and quadrupolar splittings increased as the extent of sample stretching was increased. The linewidth of the ^1^H and ^2^H NMR peaks also increased as the sample was stretched. ^2^H peak splittings and linewidths are presented in Table 1.

**Table 1:** ^2^H longitudinal (*T*_1_) and transverse (*T*_2_) nuclear spin relaxation time constants, peak splittings and average linewidths for DMSO‐*d*_6_ dissolved in gelatin gel measured at 298 K under several extents of stretching. a→d correspond to the ^2^H multiplet components labelled in Figure 1. Errors correspond to confidence intervals from least squares fitting.

Extent/cm	*T*_1_/ms				*T*_2_/ms				Splittings/Hz		Linewidths/Hz	
	a	b	c	d	a	b	c	d	outer	inner	outer	inner
0	444 ± 1				435.6 ± 0.4				0		1.25	
3*	444 ± 2	444.2 ± 0.6		450 ± 2	435 ± 4	441 ± 3		406 ± 5	−6.9	0	1.6	1.5
6	443 ± 3	445 ± 2		445 ± 4	430 ± 5	432 ± 3		422 ± 5	−21.2	−2.9	2.9	1.3
9	440 ± 4	441 ± 3	447 ± 3	436 ± 3	421 ± 23	425 ± 3	416 ± 4	418 ± 23	−48.3	−6.1	2.6	1.4
12	449 ± 5	448 ± 5	457 ± 5	456 ± 4	421 ± 8	414 ± 4	421 ± 4	425 ± 7	−73.0	−9.4	2.5	1.4

* Values measured with unresolved inner ^2^H NMR peaks.

### ^2^H Relaxation

Given the differential line broadening of the inner and outer doublet components in the ^2^H NMR spectra, relaxation time constants were measured to explore possible mechanisms that underlie this effect. ^2^H transverse (*T*_2_) and longitudinal (*T*_1_) nuclear spin relaxation time constants of DMSO‐*d*_6_ were measured using Carr‐Purcell‐Meiboom‐Gill (CPMG) and inversion recovery *rf*‐pulse sequences, respectively (see Table 1). Despite the differential linewidths observed for the two sets of doublets, the spectra revealed almost identical transverse relaxation time constants, and identical longitudinal relaxation time constants, for all peaks under the same experimental conditions (extents of stretching).

### Spectral Simulations

**Figure 3:**
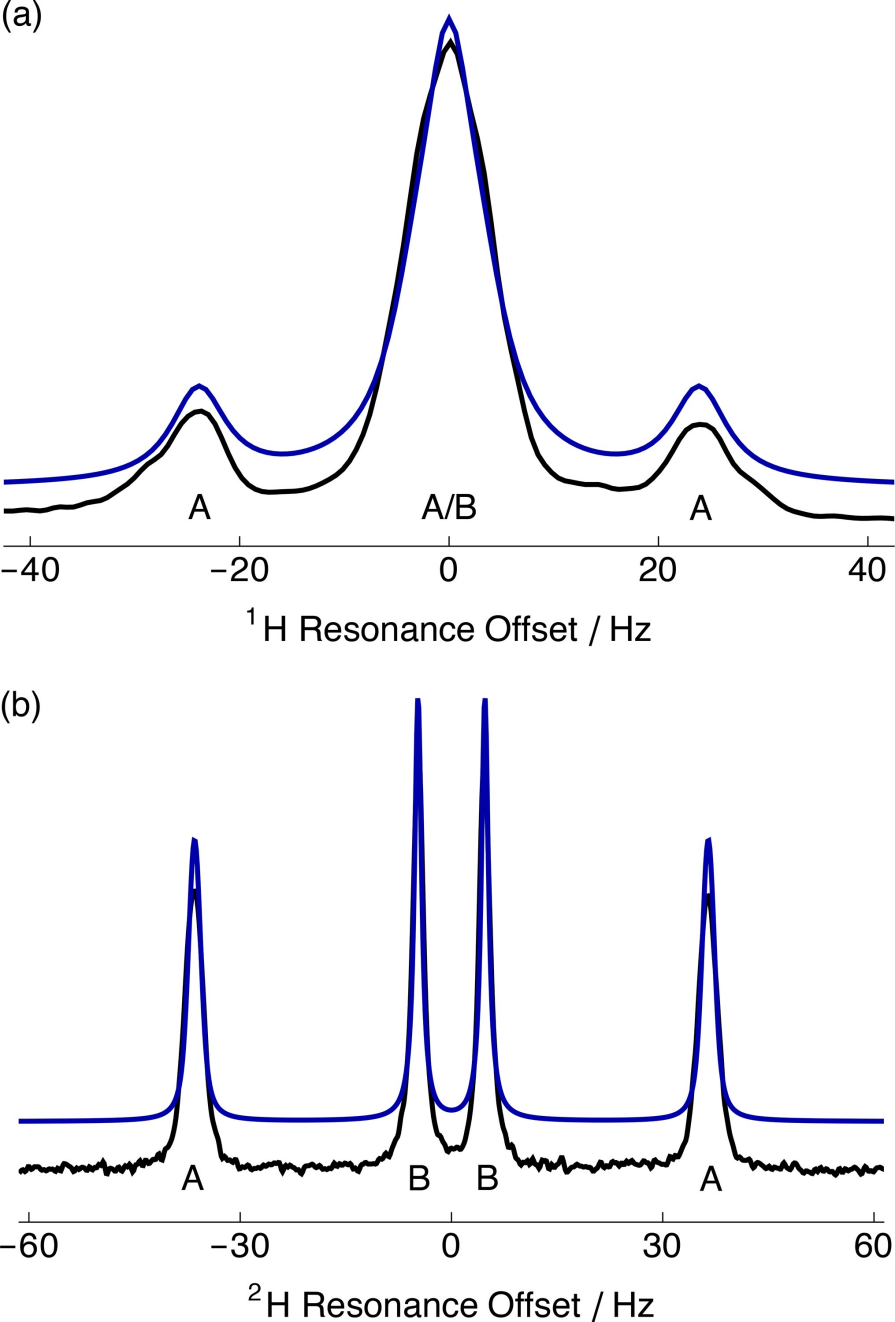
Relevant portions of the (a) ^1^H and (b) ^2^H NMR spectra of 5 % DMSO‐*h*_6_ and 5 % DMSO‐*d*_6_ dissolved in a stretched gelatin gel (extent=12 cm; 1.7‐fold) acquired at 298 K. Black line: experimental spectrum; Blue line: simulated spectrum using the *Mathematica*‐based NMR software package *SpinDynamica* with $\tau_{C}$ =38.5 ps assuming fast methyl group rotation (see Discussion).^[13]^ Peaks belonging to different enantiomers are labelled A/B.

Since the differential line broadening in Figure 2(b) cannot be explained by differences in measured transverse relaxation time constants, simulations were performed to consider the additional effect of a Gaussian distribution of S−Me order parameters. Figure 3 shows the relevant portions of the experimental (black) and simulated (blue) NMR spectra: (a) ^1^H and; (b) ^2^H of 5 % DMSO‐*h*_6_ and 5 % DMSO‐*d*_6_ dissolved in a gelatin gel that was stretched by 12 cm (1.7‐fold length change). A satisfactory agreement between experimental and simulated spectra was achieved (see Discussion).

The experimental ^1^H NMR spectrum (see Figure 3a) showed a ~1 : 6 : 1 multiplet. The outer peaks were well resolved with an RDC of $\langle \omega_{D}^{A}\rangle /2\pi$ =23.9 Hz and a full‐width at half‐maximum height (FWHM) of ~7.1 Hz. The central peak had a FWHM of ~9.8 Hz. The experimental spectrum was adequately simulated with 6 Hz FWHM Lorentzian line broadening using the following parameters: $S_{0}^{A}$ =0.77 × 10^−3^, $S_{0}^{B}$ =0.10 × 10^−3^ and $\omega_{D}/2\pi$ =−20.74 kHz.

The experimental ^2^H NMR spectrum (see Figure 3b) showed two doublets with peaks separated by $\langle \omega_{Q}^{A}\rangle /2\pi$ =−73.0 Hz and $\langle \omega_{Q}^{B}\rangle /2\pi$ =−9.4 Hz, respectively, with both doublets being well resolved. The FWHM of the inner and outer peaks were ~1.4 and ~2.5 Hz, respectively. The ratio of peak integrals was ~1 : 1 : 1 : 1. The experimental spectrum was well simulated using the following parameters: $S_{0}^{A}$ =0.77 × 10^−3^, $S_{0}^{B}$ =0.10 × 10^−3^, $\sigma^{A}$ =0.015 × 10^−3^, $\sigma^{B}$ =0.002 × 10^−3^ and $\omega_{Q}/2\pi$ =288.5 kHz.

### Stretch‐Induced Ordering

**Figure 4:**
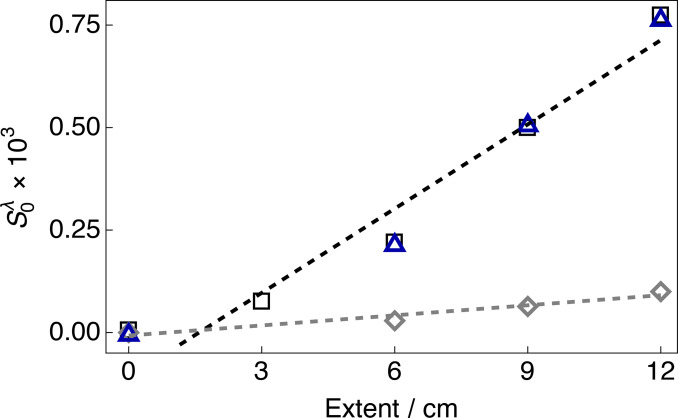
S−Me Bond order parameters $S_{0}^{\lambda }$ for the ^1^H and ^2^H nuclear spins of 5 % DMSO‐*h*_6_ and 5 % DMSO‐*d*_6_ dissolved in a gelatin gel measured at 298 K under several extents of stretching. Black squares: outer peak, ^2^H RQC; Grey diamonds, inner peak ^2^H RQC; and Blue triangles, outer peak ^1^H RDC. Data were fitted with a straight‐line function including a non‐zero intercept. Black dashed line: inner peak data (slope=(0.068±0.007)×10^−3^ cm^−1^; intercept= (−0.11±0.05)×10^−3^); Grey dashed line, inner peak data (slope=(0.008±0.001)×10^−3^ cm^−1^; intercept= (−0.01±0.01)×10^−3^).

Figure 4 shows the S−Me bond order parameter $S_{0}^{\lambda }$ for the ^1^H and ^2^H nuclear spins of 5 % DMSO‐*h*_6_ and 5 % DMSO‐*d*_6_ dissolved in the gelatin‐gel sample under several extents of stretching. Estimates of $S_{0}^{A}$ from ^1^H RDCs were in good agreement with those from ^2^H RQCs. A linear dependence of $S_{0}^{\lambda }$ on the extent of stretching was observed; *i. e*., increased stretching led to a proportional increase of S−Me bond order parameters. The data were well fitted by a straight‐line function with zero intercept. $S_{0}^{A}\;$ was found to be 0.77 × 10^−3^ for the most stretched hydrogel sample (extent=12 cm, 1.7‐fold) and was consistently larger than $S_{0}^{B}$ over the full range of stretching conditions. For samples stretched only 1.2‐fold (extent=3 cm), the inner peak splittings were no longer clearly resolved. $S_{0}^{A}=S_{0}^{B}$ =0 were assumed for relaxed gelatin gel samples (extent=0 cm).

### Slice Selective Imaging

**Figure 5:**
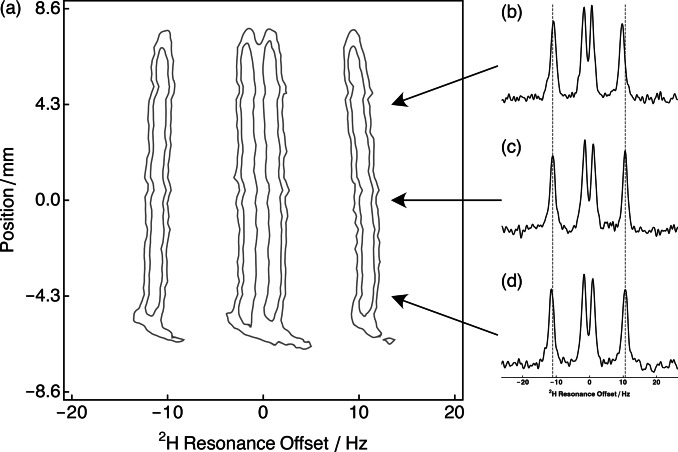
(a) Relevant portion of the slice selective ^2^H *z*‐image acquired with a slice thickness of 0.29 mm for 5 % DMSO‐*d*_6_ dissolved in a stretched gelatin gel (extent=9 cm; 1.5‐fold) acquired at 298 K. 1D spectra are shown at slice positions: (b) +4.3 mm; (c) 0 mm; and (d) −4.3 mm.

Figure 5(a) shows the relevant portion of a ^2^H *z*‐image of a sample of 5 % DMSO‐*d*_6_ dissolved in a gelatin gel that was stretched by 9 cm (1.5‐fold). Two quadrupolar doublets with different ^2^H peak splittings are evident in the image. Figs. 5(b)‐5(d) show spectra acquired from a 0.29 mm slice at positions of +4.3 mm, 0 mm and −4.3 mm from the centre of the NMR tube, respectively. Linewidths of the inner and outer doublets were much more similar than in Figure 2, highlighting an inhomogeneous contribution to the observed linewidths along the stretching direction. The RQC was smaller at the top of the tube than at the bottom.

## Discussion

### Single S‐Me Bond Order

Estimates of only single S−Me bond order parameters $S_{0}^{\lambda }$ were available in these experiments.^[22]^ DMSO has an insufficient number of ^1^H/^2^H sites to derive all elements of the symmetric part of the order matrix (three sites are required to eliminate numerical degeneracy, while one was available). More complicated probe molecules with an increased number of ^1^H/^2^H sites would be required to derive the full (symmetric) order matrix, in the case of molecular *C*_S_ symmetry.[^23^, ^24^] In anisotropic hydrogel samples, stretching is known to yield positive S−Me bond order parameters, while compression leads to negative values.^[25]^ We therefore assumed that the signs of the measured ^1^H and ^2^H splittings for methyl groups were positive and negative, respectively. The signs of residual interactions can generally be obtained from the estimates of the magnitude of ^1^H−^13^C RDCs.^[26]^ When observable in our experiments, ^13^C satellite peaks in ^1^H NMR spectra showed no change in splitting (|^1^ *J*_CH_|=139.3 Hz) upon stretching of the gelatin gel sample. The combination of RDCs and RQCs has been previously used to determine the alignment of H_2_O@C_60_ dissolved in a liquid crystal.^[27]^

### RDCs and RQCs

In the case of a dipolar coupled 3‐spin‐1/2 system, such as the CH_3_ groups of DMSO embedded in an anisotropic environment, with an alignment direction parallel to the static magnetic field **B_0_**, the ^1^H NMR spectrum splits into a triplet with a pronounced RDC.^[7]^ The three equally separated components of the methyl triplet have a splitting of:

(13) $$\left\langle \omega_{D}^{\lambda }\right\rangle =-\frac{3}{2}\omega_{D\;}S_{0}^{\lambda },$$

for enantiomer λ . Using Eq. (13) and an internuclear ^1^H−^1^H distance of *r*=179.6 pm, corresponding to a dipolar coupling constant of $\omega_{D}$ /2π =−20.74 kHz, we estimated S−Me bond order parameters for enantiomer A from the outer peak ^1^H splittings at several extents of stretching of the gelatin gel samples (see Figure 4). The triplet from enantiomer B was not resolved in the ^1^H NMR spectrum.

The electric quadrupole moment of ^2^H interacts with EFGs present at the nucleus arising from the stretched collagen molecules that make up gelatin. In this anisotropic environment, the ^2^H NMR spectrum reveals a methyl doublet split by:

(14) $$\left\langle \omega_{Q}^{\lambda }\right\rangle ≃-\frac{1}{3}\omega_{Q\;}S_{0}^{\lambda }.$$

Using Eq. (14) with the S−Me bond order parameter of $S_{0}^{A}=$ 0.77 × 10^−3^ at a stretching extent of 1.7‐fold (12 cm) and the experimentally determined value of $\langle \omega_{Q}^{A}\rangle /2\pi$ =−73.0 Hz, we calculated $\omega_{Q}/2\pi$ to be 288.5 kHz. This corresponds to $C_{Q}$ =190.3 kHz, which is close to the literature value obtained for a sp^3^‐type hybridization state of the directly bonded carbon atom.[^28^, ^29^] From this value of $\omega_{Q}$ , the S−Me bond order parameters were calculated from our experimental ^2^H NMR spectra (see Figure 4).

There was a larger deviation from the linear trend at lower extents of sample stretching (see Figure 4). Observed ^1^H dipolar and ^2^H quadrupolar splittings are weighted averages of the values in the predominantly isotropic bulk interstitial medium and the less abundant anisotropic medium. The mobile DMSO molecules sample these environments in fast exchange. A small increase in the residence time between guest DMSO and the collagen helices as the hydrogel sample is stretched would increase the apparent size of the measured RDC and RQC values.

### Enantiomer Discrimination

The chiral nature of gelatin allows enantiomers and prochiral sites to be discriminated under the anisotropic conditions of hydrogel stretching or compression. The two methyl groups covalently bound to the tetrahedral S atom of DMSO are spatially inequivalent in these systems, which manifests as a set of two triplets in the ^1^H spectrum and two doublets in the ^2^H NMR spectrum. This was clearly observed in the ^2^H NMR spectrum of DMSO dissolved in the stretched gelatin gel (see Figure 1). The ^2^H NMR spectrum of acetone‐*d*_6_, which possesses molecular *C*_2v_ symmetry, exhibited only a single quadrupolar splitting.

The resolved ^2^H quadrupolar splittings (under several extents of stretching) were different for the two discriminated pro‐R/S enantiomer doublets. A straightforward way to evaluate the enantiomer discrimination capabilities of anisotropic hydrogels is to compute the differential ordering effect (DOE) parameter:^[30]^

(15) $$DOE=2\frac{\left|\Delta \langle \omega_{Q}\rangle \right|}{\Sigma \langle \omega_{Q}\rangle },$$

where $\Delta \left\langle \omega_{Q}\right\rangle =\left|\omega_{Q}^{A}\right|+\left|\omega_{Q}^{B}\right|$ and $\Sigma \left\langle \omega_{Q}\right\rangle =\left|\omega_{Q}^{A}\right|-\left|\omega_{Q}^{B}\right|$ are the sum and difference, respectively, of the absolute values of the two ^2^H quadrupolar splittings. For hydrogel stretching extents of 1.3‐, 1.5‐, and 1.7‐fold (6, 9 and 12 cm, respectively), the DOE parameter was found to be 1.54 ± 0.02 with no significant variation between stretching extents (expected due to the linear dependence of $\left\langle \omega_{Q}^{\lambda }\right\rangle$ on the extent of sample stretching).^[2]^ This value was similar to other DOE parameters for prochiral molecules dissolved in liquid crystals;^[30]^ and the finding underscores the value of anisotropic hydrogel samples such as gelatin as suitable media for enantiomeric discrimination.

### Effective Correlation Time

Measured values of *T*_1_ were only marginally longer than *T*_2_, indicating that the nuclear spin ensemble was relaxing close to the extreme narrowing regime.^[20]^ Using Eq. (11*b*) with the quadrupolar coupling frequency of $\omega_{Q}/2\pi$ =288.5 kHz and the average value of *T*_2_=435.6 ms from a relaxed sample we estimated $\tau_{e}$ =4.3 ps; this value was confirmed using Eq. (11*a*). We surmise that the short value of $\tau_{e}$ is predominantly governed by the significantly faster rotation of the methyl group (picoseconds) rather than the correlation time of molecular tumbling in the gel (nanoseconds),^[31]^ *i. e*., the ^2^H relaxation time constants reflect the high mobility of the deuterated methyl groups. In the limit of rapid methyl group rotation, $\tau_{e}$ becomes nine times shorter than for a rigid methyl group, *i. e*., $\tau_{c}=9\tau_{e}$ =38.5 ps.^[20]^ This is equivalent to using a methyl rotor‐averaged value of $\omega_{Q}$ /3. The value of $\tau_{c}$ is similar to that found for CH_3_CN dissolved in a liquid crystal,^[32]^ but considerably shorter than previously found for ^23^Na^+^ ions under the same conditions ($\tau_{c}$ =1.5 ns).^[25]^ *T*_1_ and *T*_2_ were very similar (nearly identical) for different extents of stretching and therefore $\tau_{e}$ did not vary significantly as a function of the extent of sample anisotropy.

There is the possibility of additional motions, *e. g*., interconversion between methyl groups, that further reduce $\omega_{Q}/2\pi$ (after methyl rotor‐averaging).^[33]^ Interestingly, simultaneously solving Eqs. (11*a*) and (11*b*) leads to $\omega_{Q}/2\pi$ =37.6 kHz ($C_{Q}$ =25.1 kHz), which is significantly lower than the established literature value of $C_{Q}$ =170 kHz;[^28^, ^29^] and $\tau_{e}$ =0.25 ns, which is in line with that found for ^23^Na^+^ ions.^[25]^ This value of $\omega_{Q}/2\pi$ is further reduced by a factor of ~2.6 compared with that found via analysis of S−Me order parameters. Similar values of $\omega_{Q}/2\pi$ have been observed for acetone‐*d*_6_ in organic inclusion compounds.^[34]^

In the case that internal motions are fast, the value of $\tau_{e}$ relevant for ^2^H relaxation becomes significantly shorter than that of overall tumbling.^[35]^ The DMSO molecules hydrogen bond to the collagen fibres via the oxygen atom. Rapid rotation (or hopping) around the O=S bond axis (on a cone of approximate tetrahedral symmetry) would result in further averaging of $\omega_{Q}/2\pi$ by ~3. The experimental ^2^H NMR spectrum in Figure 3(b) is well‐reproduced using these parameters, provided $S_{0}^{\lambda }$ and $\sigma^{\lambda }$ are both increased by ~2.6. This finding highlights the potential importance of additional internal motions other than methyl group rotation on the calculated order parameters.

### ^1^H Spectral Simulations

The ^1^H NMR spectra of DMSO displayed peak integrals of ~1 : 6 : 1 at the larger extents of stretching of the gels (see Figure 3(a)) whereas for a single *J*‐coupled triplet the expected ratio of integrals would be 1 : 2 : 1. This experimental outcome implies the existence of a second, unresolved triplet that resides within the broader linewidth of the central peak. The 1 : 6 : 1 triplet can be understood as a superposition of a 1 : 2 : 1 triplet with a large RDC for one of the methyl groups and an unresolved 1 : 2 : 1 triplet with a small RDC for the other methyl group. This result is consistent with the observation of a pair of quadrupolar doublets in the ^2^H NMR spectrum. More extensive stretching of the gelatin gel, or reduced line broadening, would be required to resolve the central triplet. In this case, ^2^H NMR is more favourable than ^1^H NMR for the discrimination of enantiomers (see above).

### ^2^H Spectral Simulations

The ^2^H NMR spectra of DMSO‐*d*_6_ showed differential linewidths for the pair of quadrupolar doublets (see Figure 3(b)), despite measurements of the transverse relaxation time constants that were identical for each peak in the spectrum (see Table 1). The ^2^H NMR spectrum was well simulated using a Gaussian distribution of S−Me bond order parameters centred at $S_{0}^{\lambda }$ with a spread of $g^{\lambda }\left(x\right)$ values that was proportional to the size of $S_{0}^{\lambda }$ , *i. e*., the ratio $\sigma^{\lambda }$ /$S_{0}^{\lambda }$ was identical for each quadrupolar doublet simulated. The enantiomer with the largest $S_{0}^{\lambda }$ demonstrated the greater linewidth, confirming that the spectral linewidth observed was proportional to the extent of hydrogel stretching (see Figure 2). The different Gaussian distributions of S−Me bond order parameters required to reproduce the linewidths of the pair of ^2^H doublets is likely to be related to the spatial inequivalence of the two methyl groups (see above). Larger disparities in ^2^H linewidths have been observed for more viscous hydrogel samples.^[2]^

### Basis of ^1^H and ^2^H Spectral Differences

The ^1^H NMR spectrum of DMSO was satisfactorily simulated using 6 Hz FWHM Lorentzian line broadening (see Figure 3(a)). The increased peak width in the ^1^H NMR spectrum was attributed to homogeneous line broadening due to ^1^H−^1^H dipolar couplings within the methyl group, and to protons along the protein backbone of gelatin (denatured collagen). The linewidth of the central ^1^H peak was also broadened by the splitting of the unresolved triplet as the gel was stretched. The observation that the ^1^H linewidth increased with extent of stretching indicates a contribution from inhomogeneous broadening. The experimental ^2^H peaks were dominated by inhomogeneous broadening and only marginally broader than their simulations, indicating a minor contribution from ^2^H−^1^H dipolar couplings, which are a factor of ~6.5 weaker than ^1^H−^1^H dipolar interactions due to the lower magnetogyric ratio of ^2^H. The Gaussian distribution of S−Me bond order parameters (centred at $S_{0}^{\lambda }$ ) used to simulate the ^2^H NMR spectrum underestimated the experimental ^1^H linewidth. The linewidth of the ^1^H NMR spectrum, therefore, was governed by homogeneous line broadening, while the ^2^H NMR spectrum was predominantly inhomogenously broadened.

### Gel Inhomogeneity from Non‐Uniform Stretching

A ^2^H *z*‐imaging experiment was used to investigate sample inhomogeneity along the direction of hydrogel stretching.^[36]^ Figure 5(a) shows two quadrupolar doublets, with different ^2^H peak splittings, as a function of position within the NMR tube. The foot observed at −5.5 mm can be attributed to imperfect shimming. Alignment along the NMR tube is not entirely homogeneous as demonstrated by the outer ^2^H peak splitting, which became larger toward the bottom of NMR tube. Furthermore, similar linewidths were observed in the slices acquired at different positions along the NMR tube as shown in Figs. 5(b)‐(d). This is in contrast to the conventional non localised ^2^H NMR spectrum, which shows differential outer and inner peak linewidths. This finding can be attributed to sample inhomogeneity that results from (slightly) non‐uniform gel stretching. This inhomogeneity was modelled using a Gaussian distribution of order parameters in the simulation of the ^2^H NMR spectrum. It is possible that sample inhomogeneity also exists in the *xy*‐plane of the stretched hydrogel, but this cannot be readily probed by our experimental setup.

## Conclusions

We present a detailed analysis of the ^1^H and ^2^H NMR spectra of DMSO as a guest molecule in stretched hydrogels. The NMR spectra showed pronounced RDC and RQCs that could be used to estimate S−Me bond ordering over a wide range of sample anisotropy. ^2^H spectral broadening was related to the size of the anisotropic interaction and was imaged by slice selective NMR spectroscopy as a function of position along the NMR tube. Homogeneous broadening attributed to ^1^H−^1^H dipolar interactions dominated the ^1^H NMR spectra wherein additional unresolved multiplet structure was also present. The results are encouraging for future applications of molecular bond‐ordering measurements in anisotropic hydrogels with ^1^H and ^2^H nuclear spins in probe molecules; and beyond to more complex molecular environments such as zeolites and ordered biological tissues.

The present observations and analysis are relevant to the interpretation of *in vivo* ^2^H magnetic resonance imaging (MRI) compared to conventional ^1^H MRI. Under conditions where bond ordering is close to the magic angle, due to the orientation of tissue fibres, RQCs will become zero, and the doublet will collapse to a singlet with a narrower linewidth. This outcome suggests a potential mechanism for observing a magic angle effect, as hyperintense regions in ^2^H MRI images arise from molecular/matrix ordering within a tissue,[^37^, ^38^] in analogy with RDCs in ordered tissues observed by conventional ^1^H MRI.

## Conflict of Interests

The authors declare that they have no conflict of interests.

1

## Data Availability

Experimental data and simulation codes are available from the corresponding author upon reasonable request.
